# Supplementary material for: Early Alterations of Intra-Mural Elastic Lamellae Revealed by Synchrotron X-ray Micro-CT Exploration of Diabetic Aortas
Source: Int J Mol Sci. 2022 Mar 17;23(6):3250. doi: 10.3390/ijms23063250 (PMC8954876; doi:10.3390/ijms23063250)
Supplement: Supplementary file 1 [file ijms-23-03250-s001.zip › Figure S1.pdf]

Figure S1:

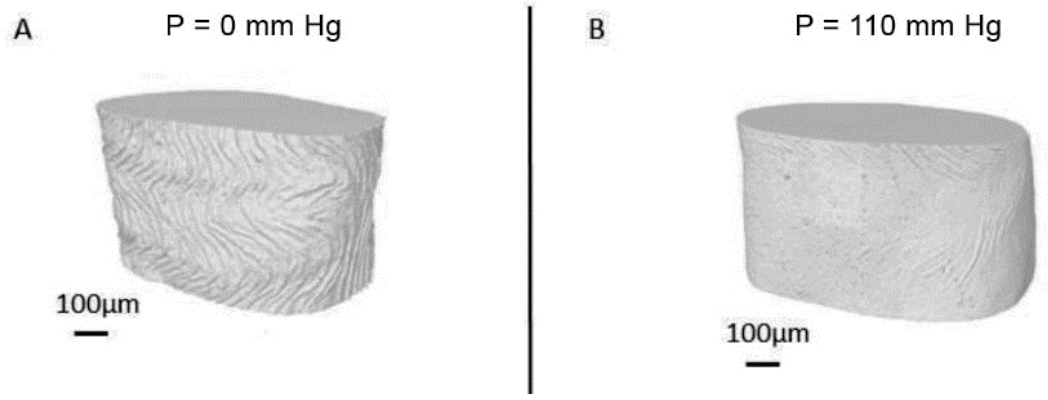

Figure S1. The effects of intra-luminal pressure on arterial tissue surface topography as viewed by X-Ray micro-CT. Segmentation methods can be used to define interfaces between discrete regions in the tomogram, therefore it was possible to depict the intimal surface in unpressurized (**A**, P = 0 mm Hg) and pressurized (**B**, P = 110 mm Hg) arteries.

Figure is from page 100 of the PhD thesis of Dr. Lucy Anne Walton (School of Medicine, Institute of cardiovascular sciences, University of Manchester, 2014). This thesis expands on the observations published in Walton et al 2015 (DOI: 10.1038/srep10074) in which pressurized and non-pressurized rat common carotid arteries were visualized by phase contrast microCT. Published with the kind courtesy of Dr. Michael J. Sheratt.
