# Supplementary material for: Early Alterations of Intra-Mural Elastic Lamellae Revealed by Synchrotron X-ray Micro-CT Exploration of Diabetic Aortas
Source: Int J Mol Sci. 2022 Mar 17;23(6):3250. doi: 10.3390/ijms23063250 (PMC8954876; doi:10.3390/ijms23063250)
Supplement: Supplementary file 1 [file ijms-23-03250-s001.zip › Figure S2.pdf]

Figure S2:

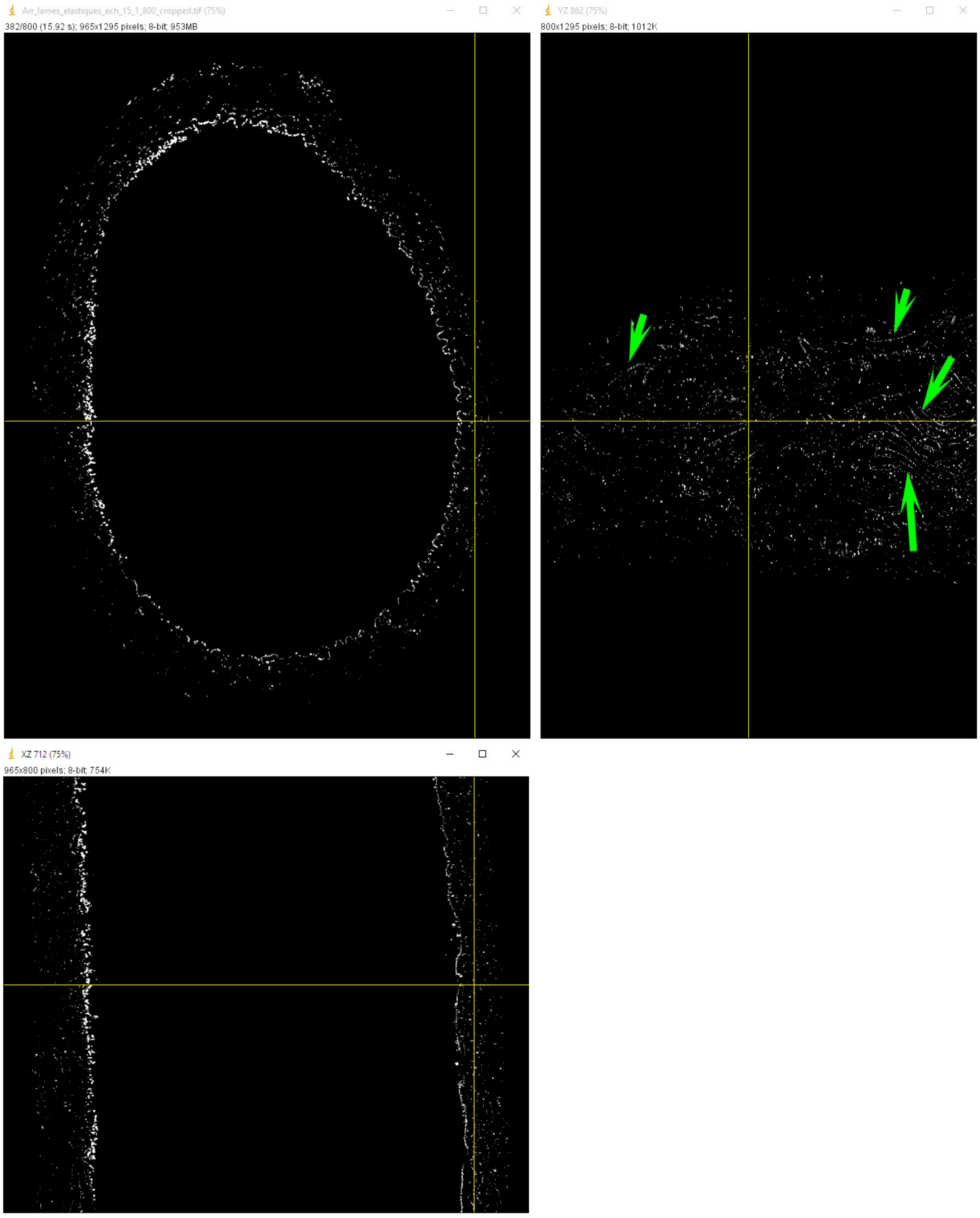

Figure S2. Orthogonal views of an 800-images stack tomogram from C57Bl6J aorta. The XY view of a 800 thresholded images (15% of the brighter pixels were kept) stack is visible (upper left). The YZ projection is located upper right and the XZ projection is lower left. Yellow lines indicate the position of the orthogonal planes. Green arrows indicate the position of filamentous structures, deep inside the medial layer.
